# Airway breathing circulation dengue: a case of multifactorial shock due to major trauma and severe dengue infection

**DOI:** 10.1186/s12245-024-00673-7

**Published:** 2024-07-16

**Authors:** Bui Hai Hoang, Thomas Vu Tang, Nguyen Dai Nghia Phan, Anh Dung Nguyen, Michael Minh Quoc Dinh

**Affiliations:** 1 Emergency and Critical Care, Hanoi Medical University Hospital, Hanoi, Vietnam; 2 Hanoi Medical University, Hanoi, Vietnam; 3 University of Tasmania, Tasmania, Australia; 4 RPA Green Light Institute, Sydney, Australia

**Keywords:** Trauma, Dengue, Major trauma, Shock

## Abstract

**Background:**

Dengue is the most common arboviral illness reported globally, endemic to most tropical and sub-tropical regions of the world. Dengue Shock Syndrome is a rare complication of severe Dengue infection resulting in haemorrhagic complications and refractory hypotension. We report on a case of severe dengue diagnosed in a patient with major trauma and illustrate some of the potential challenges and considerations in the clinical management of such cases.

**Case Presentation:**

A 49-year-old female presented following a road trauma incident with multiple abdominal injuries requiring urgent laparotomy. Her recovery in Intensive Care Unit was complicated by the development of Dengue Shock Syndrome characterised by a falling haemoglobin and platelet count, multiorgan dysfunction and prolonged hospital stay.

**Conclusions:**

Dengue Shock Syndrome may complicate fluid management and bleeding control in major trauma cases. Awareness of Dengue, particularly in endemic areas and returned travellers may help facilitate early diagnosis and management of complications.

## Introduction

Dengue is the most common arboviral illness reported globally, endemic to most tropical and sub-tropical regions of the world [1]. The Dengue flavivirus is transmitted by the *A aegypti* mosquito with an incubation period of one to two weeks. Although most dengue cases result in a mild, self-limiting febrile illness, around 5% develop severe dengue, characterised by severe vomiting, haemorrhagic manifestations (petechial rash, gastrointestinal bleeding), which can progress to dengue shock syndrome (DSS) in 1% of dengue cases. DSS is characterised by refractory hypotension and multiorgan dysfunction. with a mortality of around 25% [2].

There are no reports of intercurrent severe dengue infection complicating cases of major trauma. Such cases may become more prevalent in the coming decade as cases of Dengue continue to increase due to urbanisation, international travel and climate change[1]. We report on a case of severe dengue diagnosed in a patient with major trauma and illustrate some of the potential challenges and considerations in the clinical management of such cases.

## Case

**Fig. 1:**
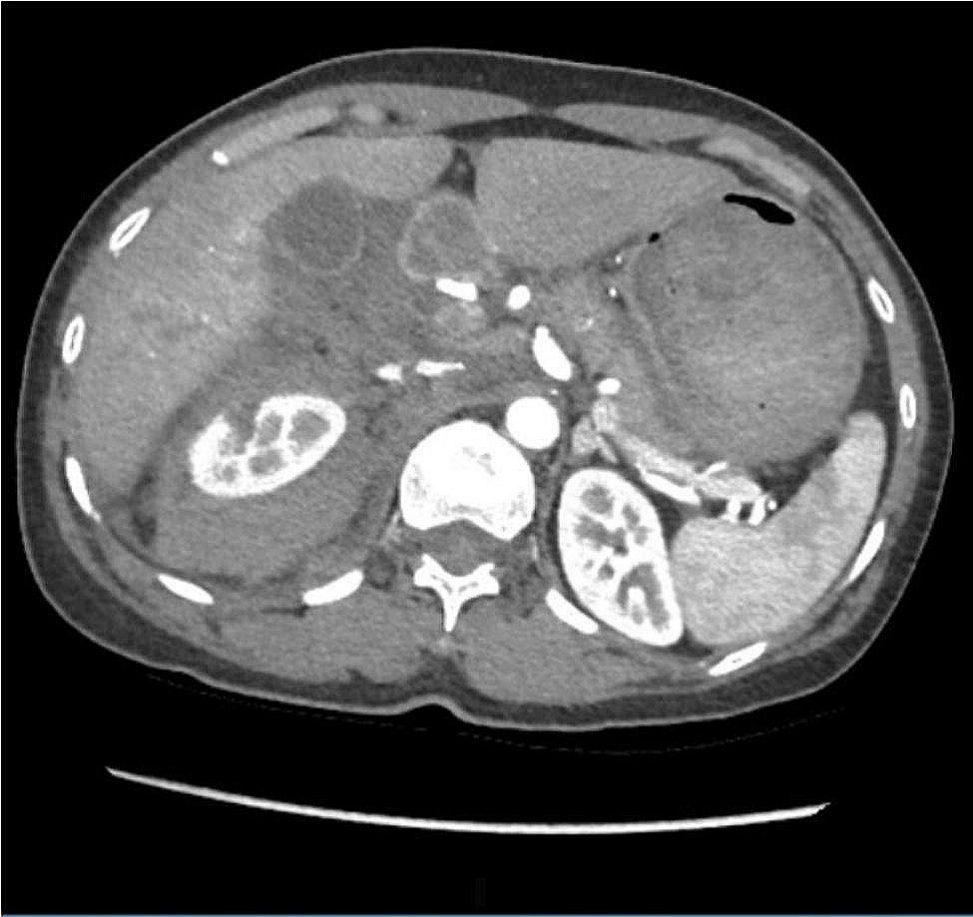
Abdominal CT (portal venous phase) imaging demonstrating high grade right kidney laceration with haemoperitoneum and liver laceration

A 49-year-old female self-presented to an emergency department of a tertiary hospital in Hanoi Vietnam with severe right flank pain after being struck by a motorcycle as a pedestrian. The speed of the motorcyclist was not known. The initial triage was ESI (Emergency Severity Index) category 2. Her medical background comprised chronic hepatitis B infection with no other known comorbidities. On examination, her Glasgow Coma Scale (GCS) was 15, blood pressure was 120/70 mmHg, oxygen saturations were 98% on room air, afebrile, respiratory rate 28 breathes/min, and heart rate 89 bpm. She had extensive bruising and tenderness to the right abdomen on examination. Within 30 min of her initial assessment, her blood pressure fell to 80/50 mmHg and pulse increased to 110 bpm. Trauma CT imaging (head, cervical spine, chest, abdomen and pelvis) revealed grade V right kidney injury, subcapsular laceration grade III liver injury (Fig. 1), fractures of ribs 10, 11, and 12 on the right, and fractures of vertebrae T12 to L2 with no clinical evidence of spinal cord injury. She did not have a head injury. Injury Severity Score was estimated to be 34.

She was given 2 L of crystalloid, 2 units of fresh frozen plasma and 2 units of packed red cells in the emergency department prior to CT imaging and before urgent transfer to the operating room for emergency trauma laparotomy with repair of descending duodenum (segment D2), formation of jejunostomy and right total nephrectomy followed postoperatively by Intensive Care Unit admission. Her liver laceration was managed conservatively.

On day 4 of admission, the patient developed a fever of 38.7 degrees Celsius which persisted to day 7, when her fever reached 40 degrees C despite prophylactic broad-spectrum antibiotics. There was no rash or gastrointestinal manifestations such as diarrhoea. During this time, her haemoglobin had dropped from 124 to 70 g/L, platelets from 368 to 62 g/L, white cell count decreased from 24.3 to 6.4 g/L, haematocrit fell from 0.38 to 0.21, serum creatinine increased from 72 to 124 umol/L and serum urea increased from 8.8 to 20.8 mmol/L. Her lactate and fibrinogen levels remained stable, changing from 3.6 to 3.1U/L and 2.43 to 3.78 g/L respectively. Blood pressure in ICU dropped from 115/60 to 90/60 mmHg on day 7 of admission.

Dengue was suspected once she developed fever because of a concurrent seasonal outbreak of Dengue in Hanoi at the time and her NS1 antigen test returned positive for Dengue virus on day 4 post operation. Serum IgM and IgG levels for Dengue were also positive. There was no history of prior history of Dengue vaccination. On day 7 she was diagnosed with DSS. Lower limb ultrasound demonstrated bilateral deep vein thromboses on Day 8. Blood cultures on the same day were positive for Acinetobacter calcoaceticus-baumannii complex which was resistant to ceftazidime and meropenem but susceptible to trimethoprim-sulfamethoxazole, which was used in this case. Progress CT imaging revealed no further intraabdominal bleeding. Her hypotension was responsive to noradrenaline, which was maintained at 0.15 microg/kg/min. Platelet and pack cell transfusion was commenced, however she developed ascites and pulmonary oedema on day 10 which was treated with a trial of frusemide but subsequently became anuric on day 12 requiring continuous venovenous hemofiltration. Her ICU stay was further complicated by Candida Albicans fungemia which was treated with voriconazole. Her renal function and urine output eventually normalised on day 14 and she was extubated on day 19 of admission and eventually discharged home on day 30.

## Discussion

The differential diagnoses of shock during the acute and perioperative phase of major trauma in the context of this case were broad and multifactorial, including ongoing haemorrhage, sepsis due to intraabdominal collections or nosocomial pneumonia, systemic inflammatory response syndrome, Disseminated Intravascular Coagulopathy, fat embolism and concurrent infections [3]. In this instance, the patient developed DSS complicated by multiorgan dysfunction and sepsis which complicated her recovery.

DSS may affect injury management and complicate recovery on a number of levels. Firstly, diagnosis of DSS may be delayed leading to inappropriate diagnosis of post operative haemorrhage and inappropriate fluid resuscitation. This was an important consideration in this patient whose blood pressure and haemoglobin dropped postoperatively coinciding with the onset of severe dengue. It was not known when the patient contracted the infection, however, with an incubation of around a week, this likely occurred prior to hospital admission. Secondly, any fluid resuscitation for presumed bleeding may further exacerbate intravascular fluid shifts leading to ascites and pulmonary oedema. Thirdly, thrombocytopaenia may exacerbate perioperative and postoperative bleeding and reduce rates of successful non-operative management of solid organ injuries such as liver lacerations observed in this case [4]. Fourthly, the relative neutropenia that may develop as part of DSS may increase susceptibility to nosocomial infections. All these factors can confound the management of complex polytrauma cases.

The pathogenesis of DSS involves increased vascular permeability, thrombocytopaenia and leukopenia which can complicate ongoing recovery from major trauma. Hypovolemic shock seen in DSS results due to gastrointestinal losses due to vomiting or bloody stools associated with severe dengue in addition to fluid shifts, ongoing bleeding and multiorgan dysfunction [5]. This patient faced several challenges with respect to fluid management. Initially, she required management of haemorrhagic shock secondary to major trauma. However, with the onset of DSS, judicious administration of intravenous fluids in conjunction with vasopressor support were required to manage third spacing fluid shifts and pulmonary oedema. Our patient tested positive for both Dengue IgM and IgG, suggesting previous infection. It is known that previous Dengue Infection increases the risk of severe dengue with subsequent infections with a different serotype of Dengue virus [2]. Infection with a different serotype of Dengue virus in secondary infection was thought to result in highly specific antibodies that can bind to the virus but lack neutralising capacity [1]. These antibodies trigger Fc-gamma receptor-mediated antibody-dependent enhancement (ADE) and a cytokine storm, resulting in more severe illness [1]. The patient’s clinical presentation is supportive of this mechanism, having developed severe illness with a background of prior infection.

## Conclusion

We report a rare case of multi-factorial shock, involving both major trauma and concurrent severe dengue infection. Judicious fluid management, vasopressor and renal support, and diligence in suspecting concurrent infection in such cases is important in achieving good patient outcomes. Dengue complicating acute injury should be considered in returned travellers and those living in endemic areas.

## Data Availability

No datasets were generated or analysed during the current study.
